# X-shaped DNA potentiates therapeutic efficacy in colitis-associated colon cancer through dual activation of TLR9 and inflammasomes

**DOI:** 10.1186/s12943-015-0369-2

**Published:** 2015-05-15

**Authors:** Jung Eun Koo, Seung Won Shin, Soong Ho Um, Joo Young Lee

**Affiliations:** Integrated Research Institute of Pharmaceutical Sciences, College of Pharmacy, The Catholic University of Korea, 420-743 Bucheon, Republic of Korea; School of Chemical Engineering and SKKU Advanced Institute of Nanotechnology (SAINT), Sungkyunkwan University, 440-746 Suwon, Republic of Korea

**Keywords:** Immunostimulatory DNA, Pattern recognition receptor, Colon cancer, Caspase-1, Dendritic cells, Immune adjuvant

## Abstract

**Background:**

Immunotherapy has been extensively pursed as a promising strategy for the treatment of cancer. Pattern-recognition receptors (PRRs) play important roles in triggering activation of innate and adaptive immunity. Therefore, agents that stimulate PRRs could be useful for cancer immunotherapy. We developed two kinds of X-shaped double-stranded oligodeoxynucleotides (X-DNA), a single unit of X-DNA (X_S_-DNA) composed of four strands of DNA and a ligated X-DNA complex (X_L_-DNA) formed by crosslinking each X_S_-DNA to the other, and investigated if they had immunostimulatory activity and could be applied to anti-cancer immunotherapy.

**Methods:**

Activation of MAPKs and NF-κB was determined by immunoblotting in bone marrow-derived primary dendritic cells (BMDCs). Immune cytokines and co-stimulatory molecules were measured by ELISA and flow cytometry analysis. Anti-cancer efficacy was examined in an azoxymethane/dextran sulfate sodium-induced colitis-associated colon cancer mouse model. Association of X-DNA and TLR9 was determined by co-immunoprecipitation followed by immunoblotting. The involvement of TLR9 and inflammasomes was determined using TLR9- or caspase-1-deficient BMDCs. Inflammasome activation was examined by degradation of pro-caspase-1 to caspase-1 and cleavage of pro-IL-1β to IL-1β in BMDCs.

**Results:**

X_L_-DNA and X_S_-DNA induced activation of MAPKs and NF-κB and production of immune cytokines and co-stimulatory molecules in BMDCs. BMDCs stimulated by X_L_-DNA induced differentiation of naïve CD4^+^ T cells to T_H_1 cells. Intravenous injection of X_L_-DNA into mice resulted in increased serum IFN-γ and IL-12 levels, showing *in vivo* efficacy of X_L_-DNA to activate T_H_1 cells and dendritic cells. X_L_-DNA greatly enhanced the therapeutic efficacy of doxorubicin, an anti-cancer drug, in colitis-associated colon cancer. X_L_-DNA directly associated with TLR9. In addition, immunostimulatory activities of X-DNA were abolished in TLR9-deficient dendritic cells. Furthermore, X-DNA induced caspase-1 degradation and IL-1β secretion in BMDCs, which were abolished in caspase-1-deficient cells.

**Conclusions:**

X-DNA induced the activation of dendritic cells as shown by the expression of immune-cytokines and co-stimulatory molecules, resulting in the differentiation of T_H_1 cells, mediated through dual activation of TLR9 and inflammasomes. X-DNA represents a promising immune adjuvant that can enhance the therapeutic efficacy of anti-cancer drugs by activating PRRs.

**Electronic supplementary material:**

The online version of this article (doi:10.1186/s12943-015-0369-2) contains supplementary material, which is available to authorized users.

## Background

Certain tumor cells, including lymphoma, skin, and cervical tumors, survive the host defense system by evading immune surveillance [1,2]. Cancer immunotherapy strengthens the host immune system to fight against cancer, making it a promising approach for cancer treatment. Pattern recognition receptors such as Toll-like receptors (TLRs) expressed on innate immune cells initiate immune responses by activating innate and adaptive immune cells through recognition of pathogen-associated molecular patterns (PAMPs) derived from infectious bacteria and viruses [3]. In addition to PAMPs, danger signals, namely damage-associated molecular patterns (DAMPs) derived from damaged cells and tissues, activate PRRs to trigger immune responses for proper repair processes [4,5]. In particular, PRRs respond to endogenous substances from tumor cells and stress ligands expressed on the surface of tumor cells, activating host immune responses as a protective defense mechanism against the developing tumor [6,7]. The expression of co-stimulatory molecules and immune cytokines in innate immune cells is critical to induce the activation of adaptive immune cells [3]. Co-stimulatory molecules interact with CD28 that is constitutively expressed on the surface of naïve CD4 positive T cells, leading to T cell proliferation and the production of cytokines and adhesion molecules [8]. IL-12 secreted by antigen presenting cells induces differentiation of naïve CD4 positive T cells (T_H_ cells) to T_H_1 effector cells. T_H_1 cells enhance the cytolytic functions of cytotoxic T cells and natural killer cells (NK cells) [9]. In addition, IFN-γ produced by T_H_1, cytotoxic T, and NK cells exerts antiviral, immune-regulatory, and anti-tumor properties [10]. Therefore, activation of the innate and adaptive immune systems has become the focus of effective strategies for cancer immunotherapy.

Development of immunostimulatory DNA assemblies has increased the possibility for pharmaceutical and biomedical applications and drawn a great deal of attention for the development of cancer immunotherapy [11]. CpG oligodeoxynucleotides (CpG ODN) have been actively explored for therapeutic purposes due to their immunostimulatory activity [12]. CpG ODN is a short single-stranded synthetic DNA molecule that contains a cytosine triphosphate deoxynucleotide followed by a guanine triphosphate deoxynucleotide. CpG ODN activates TLR9, inducing the expression of immune cytokines and stimulating innate and adaptive immune responses [13]. TLR9 is a type I transmembrane receptor localized on endosomes, thereby recognizing DNA with CpG motifs derived from phagocytized bacteria and viruses. Understanding of the TLR9 signal cascade has prompted the clinical development of TLR9 agonists to treat cancer as well as infectious diseases, asthma, and allergies [12]. TLR9 agonists such as CpG ODN enhance antitumor T-cell responses when used as an adjuvant for anti-cancer therapy [14]. There have been attempts to develop more efficient immunostimulatory DNA. Roberts et al. reported that longer DNA molecules are taken up more efficiently by cells than shorter ones [15]. Immunostimulatory activity is enhanced by complexing CpG ODN into a Y-shaped form compared with single-stranded or double-stranded ODN [16]. Furthermore, highly structured double-stranded DNA can be useful as a drug delivery system. For example, doxorubicin, an anti-cancer drug, can be intercalated into plasmid DNA and delivered to metastatic colonies of colon carcinoma cells in the mouse liver [17,18]. Thus, the development of newly designed DNA assemblies may extend potential pharmaceutical applications.

We previously described the development of X-shaped double-stranded oligodeoxynucleotides (X-DNA) for possible biomedical applications [11]. A single unit of the X-DNA (X_S_-DNA) has an X-shaped structure composed of four strands of oligodeoxynucleotides. We also constructed a ligated X-DNA complex (X_L_-DNA) by crosslinking X-DNAs by introducing a complementary ACGT sequence at the end of each strand (Additional file 1: Figure S1) [11]. We investigated whether X_L_-DNA and X_S_-DNA have immunostimulatory activity and attempted to find possible pharmaceutical applications for cancer immunotherapy. Both X_S_-DNA and X_L_-DNA induced the production of immune cytokines and co-stimulatory molecules in dendritic cells, and the X_L_-DNA was more potent. X_L_-DNA treatment of dendritic cells in culture and to mice via intravenous injection led to the differentiation of naïve CD4^+^ T cells to T_H_1 cells. Combination therapy with X_L_-DNA greatly enhanced the anti-cancer efficacy of doxorubicin in a mouse model of colitis-associated colon cancer. The immunostimulatory activity of X_L_-DNA was mediated through TLR9 and inflammasomes. The results indicate a promising role for X-DNA as an immune activator in cancer immunotherapy.

## Results

### X-DNA induces the activation of bone marrow-derived primary dendritic cells

**Figure 1:**
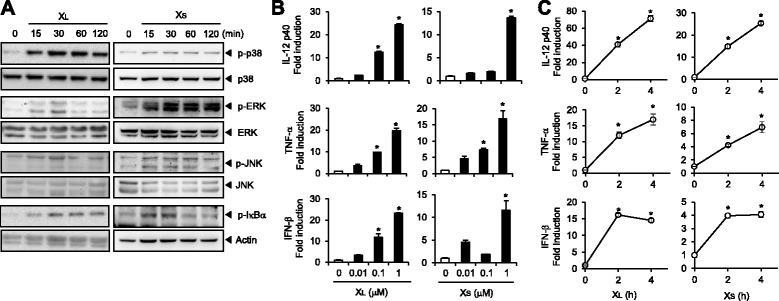
X_L_-DNA and X_S_-DNA induce the activation of MAPKs and NF-κB and increase cytokine mRNA levels in bone marrow-derived primary dendritic cells. **A**. BMDCs were treated with X_L_-DNA (1 μM) or X_S_-DNA (1 μM) for the indicated time periods. Cell lysates were analyzed by immunoblotting. **B**, **C**. BMDCs were treated with (B) X_L_-DNA or X_S_-DNA at the indicated concentrations for 4 h or (C) 1 μM of X_L_-DNA or X_S_-DNA for the indicated time periods. Cytokine mRNA levels were measured by quantitative real time-PCR analysis and normalized with β-actin. The mRNA level of each cytokine was expressed as fold induction compared with control. Values are mean ± SEM (n = 3). *, Significantly different from control (*p* < 0.05). Representative data from three independent experiments are presented.

**Figure 2:**
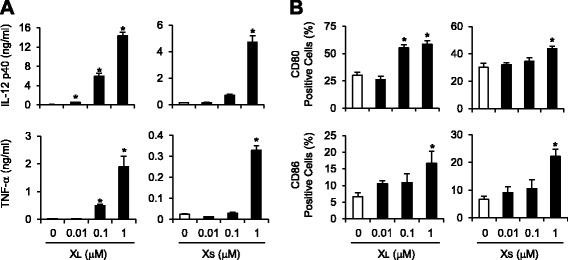
X_L_-DNA and X_S_-DNA induce the expression of cytokines and co-stimulatory proteins in bone marrow-derived primary dendritic cells. **A**. BMDCs were treated with X_L_-DNA or X_S_-DNA at the indicated concentrations for 18 h. Culture media were collected and analyzed for each cytokine by ELISA. **B**. After BMDCs were stimulated with X_L_-DNA or X_S_-DNA for 24 h, the surface expression of CD80 and CD86 was analyzed by flow cytometry. Values are mean ± SEM (n = 3). *, Significantly different from control (*p* < 0.05). Representative data from three independent experiments are presented.

To investigate the immunostimulatory potential of X-DNA, we examined whether X_L_-DNA and X_S_-DNA could induce the activation of dendritic cells that are the representative innate immune cells. The phosphorylation of p38, ERK, JNK, and IκBα was induced by X_L_-DNA, a ligated complex form of X-DNA, in bone marrow-derived primary dendritic cells (BMDCs) (Figure 1A) showing that X_L_-DNA activated MAPKs and NF-κB pathways. Similarly, X_S_-DNA was also able to induce the phosphorylation of p38, ERK, JNK, and IκBα in BMDCs (Figure 1A). X_L_-DNA or X_S_-DNA treatment increased mRNA levels of IL-12, TNF-α, and IFN-β in time- and dose-dependent manners in dendritic cells (Figure 1B and C). Secreted protein levels of IL-12 and TNF-α were also increased by X_L_-DNA or X_S_-DNA in dendritic cells (Figure 2A). In addition, co-stimulatory molecules such as CD80 and CD86 were upregulated by X_L_-DNA or X_S_-DNA treatment (Figure 2B). Overall, the potency in immune-stimulation was greater with X_L_-DNA than with X_S_-DNA.

These results demonstrate that X_L_-DNA and X_S_-DNA stimulated immune activity in dendritic cells, inducing the expression of immune-cytokines and co-stimulatory molecules.

### Activation of dendritic cells by X-DNA results in activation of a T_H_1 response

**Figure 3:**
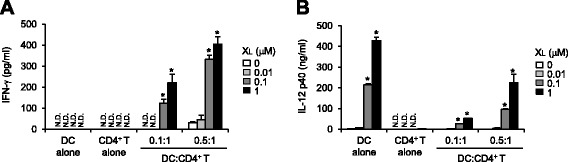
Dendritic cells activated by X_L_-DNA induce the differentiation of naïve CD4^+^ T cells to T_H_1- effector cells. BMDCs were treated with ovalbumin peptide (323-339) (5 μg/ml) in the presence of X_L_-DNA with indicated concentrations for 24 h, then further co-cultured with naïve CD4^+^ T cells for 5 days. Levels of **(A)** IFN-γ and **(B)** IL-12 p40 in culture supernatants were measured by ELISA. Values are mean ± SEM (n = 3). *, Significantly different from X_L_-DNA untreated group (*p* < 0.05). Representative data from three independent experiments are presented. N.D., not detected. DC, dendritic cells.

Co-stimulatory molecules expressed on antigen-presenting cells prompt the differentiation of CD4^+^ T cells. In particular, IL-12 skews the differentiation of naïve CD4^+^ T cells to a T_H_1-cell lineage. Therefore, we investigated whether dendritic cells activated by X_L_-DNA were able to differentiate naïve CD4^+^ T cells to T_H_1 effector cells. After BMDCs were treated with ovalbumin peptide (323-339) in the presence of X_L_-DNA, the cells were co-cultured with naïve CD4^+^ T cells for 5 days. The protein levels of IFN-γ and IL-12 were determined from cell culture supernatants. The secretion of IFN-γ, which is a surrogate marker for T_H_1 cell differentiation, was up-regulated by BMDCs treated with X_L_-DNA (Figure 3A) indicating the differentiation of naïve CD4^+^ T cells to T_H_1 cells. Addition of more dendritic cells resulted in higher production of IFN-γ, indicating the dependency of IFN-γ production by T cells on dendritic cells. However, the production of IL-4, which is the hallmark of T_H_2 cell differentiation, was not evident (data not shown). The levels of IL-12 were consistently elevated in both dendritic cells alone and dendritic cells plus naïve CD4^+^ T cells in an X_L_-DNA dose-dependent manner (Figure 3B), confirming IL-12 production by dendritic cells in this co-incubation system.

**Figure 4:**
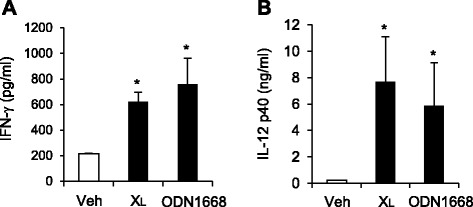
X_L_-DNA induced T_H_1 differentiation *in vivo*. X_L_-DNA (20 nmole) or ODN1668 (20 nmole) was given via intraperitoneal injection to mice. After 3 h, serum was obtained. The serum concentrations of **(A)** IFN-γ and **(B)** IL-12 p40 were determined by ELISA. Values are mean ± SEM (n = 4). *, Significantly different from vehicle (*p* < 0.05). Representative data from two independent experiments are presented. Veh, vehicle.

We further investigated whether X_L_-DNA induced T_H_1 differentiation *in vivo*. X_L_-DNA was given via intraperitoneal injection to mice. Blood levels of IFN-γ and IL-12 were enhanced by X_L_-DNA injection (Figure 4A and B), demonstrating the activation of T_H_1 cells and dendritic cells, respectively. The observed potency to increase IFN-γ and IL-12 was comparable with that of ODN1668, a well-known immunostimulatory oligonucleotide. The results suggest that X_L_-DNA has *in vivo* immunostimulatory activities to activate innate immune cells to produce IL-12 and to differentiate naïve CD4^+^ T cells to T_H_1 cells.

### X_L_-DNA enhances the efficacy of anti-cancer drug therapy

**Figure 5:**
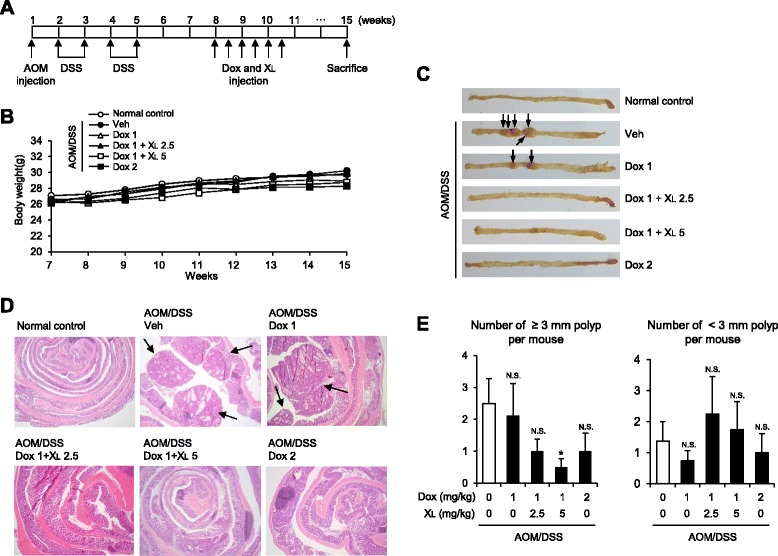
X_L_-DNA enhances anti-cancer efficacy of doxorubicin in the AOM/DSS-induced colonic cancer model. **A**. Two cycles of dextran sodium sulfate (DSS) treatment in combination with azoxymethane (AOM) resulted in more than 83% incidence of colonic neoplasms that were most frequently observed in the middle and distal colon. Eight weeks after AOM injection, doxorubicin (Dox, 1 or 2 mg/kg) and/or X_L_-DNA (2.5 or 5 mg/kg) were i.v injected. Each group had twelve mice except for the normal control group, which had ten mice. **B**. Changes in mouse body weight were monitored. **C-E**. Colons were obtained 15 weeks after AOM injection. **C**. Macroscopic features of colon. **D**. H&E staining of colon sections. Arrow indicates tumor. Magnification: 40x. **E**. The number of colonic polyps ≥3 mm or <3 mm in size (n = 8). The incidence of colonic polyp formation was zero in the normal control group (not shown in figure). Values are mean ± SEM (n = 8). *, Significantly different from AOM/DSS alone group (*p* < 0.05). N.S., not significant from AOM/DSS alone group. Veh, vehicle.

An enhanced host immune response would be beneficial for elimination of tumor cells. Therefore, the use of immune adjuvants that promote T_H_1 cell responses offers an efficient strategy for anti-cancer therapy. Since X_L_-DNA has immunostimulatory activity, we examined whether X_L_-DNA could be applied to anti-cancer therapeutics using a mouse colitis-associated colorectal cancer model with doxorubicin as the anti-cancer drug. Dextran sulfate sodium treatment in combination with azoxymethane injection resulted in an incidence of colonic polyps formation of more than 83% (10 of 12 mice) (Figure 5A). Doxorubicin with or without X_L_-DNA was intravenously injected six times from the eighth to the tenth week, and the body weights of mice were monitored. Overall, doxorubicin-treated mice showed a slight decrease in body weight during the therapeutic period (Figure 5B). Tumors were formed in the colons of AOM/DSS-treated mice (Figure 5C). While 1 mg/kg of doxorubicin was not sufficient to abolish tumor formation induced by AOM/DSS treatment, tumor formation was not observed in mice treated with a combination therapy of 1 mg/kg doxorubicin with 2.5 or 5 mg/kg X_L_-DNA (Figure 5C). The therapeutic potency of 1 mg/kg doxorubicin was greatly enhanced by combination with X_L_-DNA, with an efficacy similar to twice the dosage of doxorubicin (Figure 5C). Histological assessments consistently showed that X_L_-DNA increased the therapeutic efficacy of doxorubicin (Figure 5D). In particular, the number of polyps greater than 3 mm size was significantly reduced by co-treatment with X_L_-DNA (Figure 5E). These results suggest the beneficial application of X_L_-DNA to enhance the therapeutic efficacy of anti-cancer drugs.

### Endocytosis of X_L_-DNA is required for its immunostimulatory activity

**Figure 6:**
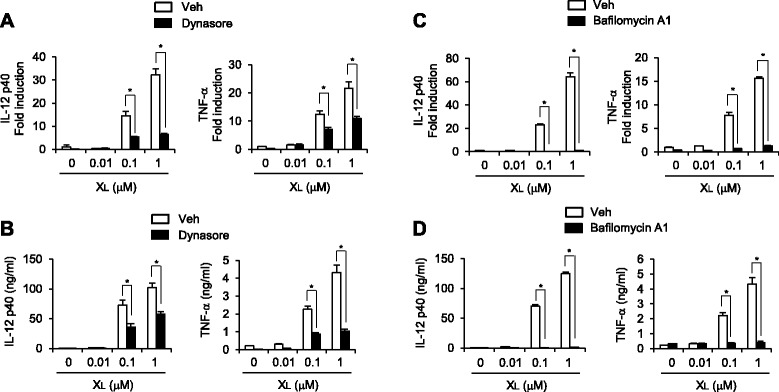
Activation of dendritic cells by X_L_-DNA is mediated through the endocytosis pathway. **A**, **B**. BMDCs were pre-treated with dynasore (40 μM) for 30 min and then treated with X_L_-DNA for (A) 4 h or (B) 18 h. **C**, **D**. BMDCs were pre-treated with bafilomycin A1 (10 nM) for 30 min and then treated with X_L_-DNA for 4 h (C) or 18 h (D). For A and C, mRNA levels were determined by quantitative real time-PCR. For B and D, concentrations of IL-12 p40 and TNF-α were measured by ELISA. Values are mean ± SEM (n = 3). *, *p* < 0.05. Representative data from three independent experiments are presented.

To investigate the mechanism behind the immunostimulatory action of X_L_-DNA, we examined whether endocytosis of X_L_-DNA was required to induce an immune response in dendritic cells. We treated BMDCs with an endocytosis inhibitor, dynasore, prior to X_L_-DNA treatment. The induction of mRNA expression of IL-12 and TNF-α by X_L_-DNA was significantly inhibited by dynasore (Figure 6A). In addition, protein levels of IL-12 and TNF-α elevated by X_L_-DNA were reduced by dynasore (Figure 6B). We also tested whether endosomal acidification was necessary for the immune activity of X_L_-DNA. Bafilomycin A1 suppressed the expression of IL-12 and TNF-α mRNA and proteins increased by X_L_-DNA in dendritic cells (Figure 6C and D). These results show that X_L_-DNA is endocytosed by immune cells to exhibit immunostimulatory activities.

### Immunostimulatory activity of X_L_-DNA is mediated through TLR9

**Figure 7:**
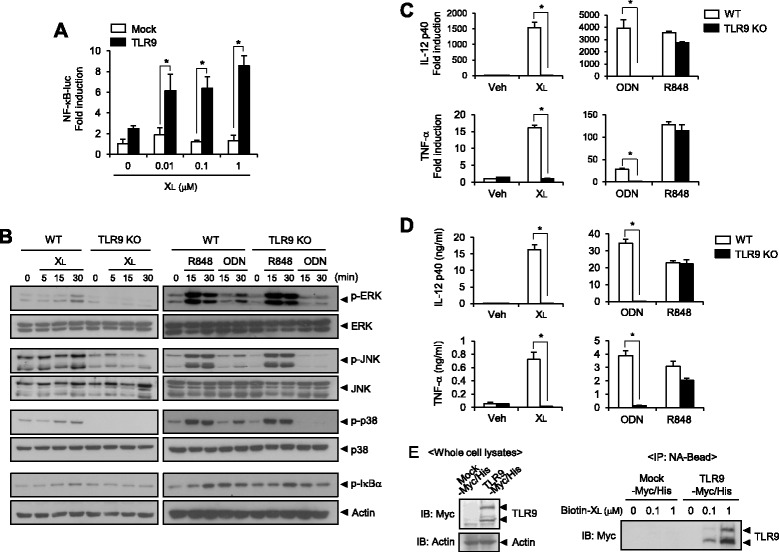
X_L_-DNA activates TLR9. **A**. After HEK293T cells were transfected with NF-κB-luc and pMSCVpuro-mTLR9-Myc-expressing plasmid, cells were treated with X_L_-DNA. Cell lysates were assayed for luciferase activities. Values are mean ± SEM (n = 3). *, *p* < 0.05. Representative data from three independent experiments are presented. **B**. BMDCs isolated from wild-type (WT) or TLR9-knockout (KO) mice were treated with X_L_-DNA (1 μM), R848 (100 ng/ml), or ODN1668 (ODN; 1 μM). Cell lysates were analyzed for immunoblotting. **C**, **D**. BMDCs from WT or TLR9KO mice were treated with X_L_-DNA (1 μM), R848 (100 ng/ml), or ODN1668 (ODN; 1 μM) for (C) 4 h and (D) 18 h. For C, mRNA levels were determined by quantitative real time-PCR. For D, concentrations of cytokines were measured by ELISA. For A, C, and D, values are mean ± SEM (n = 3). *, *p* < 0.05. **E**. Whole cell lysates were obtained from HEK293T cells transfected with mTLR9-Myc/His-expressing plasmid or empty vector and were incubated with biotinylated X_L_-DNA for 4 h followed by incubation with NA-beads for 2 h. Co-precipitated proteins were analyzed by immunoblotting with anti-Myc antibody. For B-E, representative data from two independent experiments are presented. Veh, vehicle.

TLR9 is expressed in the endoplasmic reticulum under basal conditions and traffics to the endosomal compartment, where it recognizes endocytosed nucleic acids [19]. Since endocytosis was required for X_L_-DNA-mediated immunostimulatory activity, we investigated whether X_L_-DNA was able to activate TLR9. As a gain-of-function study, HEK293T cells were transfected with TLR9-expressing plasmid together with the NF-κB-luciferase reporter gene, and then treated with X_L_-DNA at the indicated concentrations. Overexpression of TLR9 conferred the responsiveness of cells to X_L_-DNA, leading to an increase in NF-κB-dependent luciferase activity (Figure 7A).

To confirm that immune cell activation by X_L_-DNA was mediated through TLR9, we examined the activity of X_L_-DNA in dendritic cells isolated from TLR9-deficient mice. X_L_-DNA lost its ability to induce phosphorylation of MAPKs such as ERK, JNK, and p38 in addition to phosphorylation of IκBα in TLR9-deficient dendritic cells (Figure 7B). X_L_-DNA-induced production of cytokines IL-12 and TNF-α was abolished in TLR9-deficient dendritic cells at both the mRNA and protein levels (Figure 7C and D). R848, a TLR7 ligand, was used as a negative control and CpG1668, a synthetic ligand of TLR9, was used as a positive control (Figure 7B, C, and D). Similarly, X_S_-DNA-induced production of IL-12 and TNF-α was abolished in TLR9-deficient dendritic cells at both the mRNA and protein levels (Additional file 2: Figure S2A and B).

To confirm whether X_L_-DNA directly bound to TLR9, an in vitro binding assay was performed. Myc/His-tagged TLR9 was expressed in HEK293T cells (Figure 7E, left panel). The cell lysates were incubated with biotin-X_L_-DNA and immunoprecipitated with NA beads followed by immunoblotting to detect TLR9 co-precipitated with X_L_-DNA (Figure 7E, right panel). TLR9 was co-precipitated with X_L_-DNA in an X_L_-DNA dose-dependent manner (Figure 7E, right panel) showing the direct association of X_L_-DNA and TLR9.

These results show that X_L_-DNA associates with TLR9, thereby exerting immunostimulatory activities.

### X-DNA activates a cytosolic inflammasome complex

**Figure 8:**
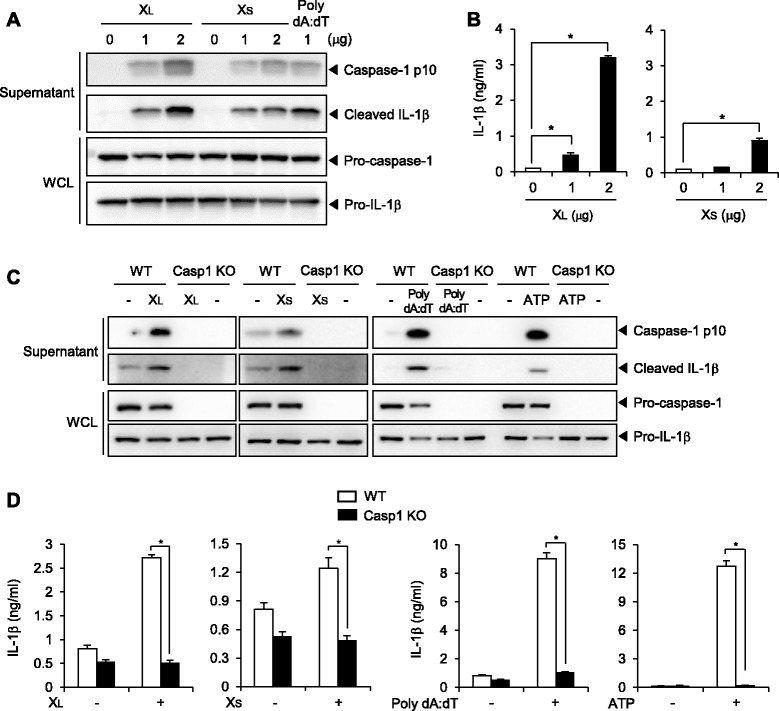
X_L_-DNA and X_S_-DNA induce inflammasome activation. **A**, **B**. After BMDCs were primed with LPS (10 ng/ml, 18 h), cells were transfected with X_L_- DNA, X_S_-DNA, or poly dA:dT for (A) 6 h or (B) 18 h. **C**, **D**. After BMDCs from wild-type (WT) or caspase-1-knockout (Casp1KO) mice were primed with LPS (10 ng/ml, 18 h), cells were transfected with X-DNA (2 μg) or poly dA:dT (1 μg) for (C) 6 h or (D) 18 h. ATP (5 mM) was added to dendritic cells primed with LPS for 1 h (C) or 18 h (D). For A and C, supernatant proteins and whole cell lysates (WCL) were loaded on gels and subjected to SDS-PAGE. For B and D, concentration of IL-1β was measured by ELISA. Values are mean ± SEM (n = 3). *, *p* < 0.05. Representative data from three independent experiments are presented.

Recently, cytosolic PRRs have emerged as targets of immune therapy. Among them, the NOD-like receptor family-dependent caspase-1 activation complex, a so called inflammasome, recognizes infectious bacteria or endogenous stimulatory substances in the cytosolic region and forms a caspase-1 activating platform to secret mature IL-1β. Therefore, we investigated if X-DNA had the ability to activate the inflammasome complex. X_L_-DNA or X_S_-DNA was transfected to BMDCs to be introduced into the cytosol. Inflammasome activation was assessed by secretion of caspase-1 p10 and cleaved IL-1β into the cell supernatant by western blotting and ELISA. Poly dA:dT was used as a positive control as it is well known to activate the inflammasome complex when transfected. Both X_L_-DNA and X_S_-DNA induced secretion of caspase-1 p10 and cleaved IL-1β in a dose-dependent manner, with X_L_-DNA being more potent (Figure 8A). Stimulation of IL-1β secretion by X_L_-DNA and X_S_-DNA was confirmed by ELISA (Figure 8B). To further confirm the activation of the inflammasome complex by X-DNA, we investigated whether inflammasome activation induced by X-DNA was dependent on caspase-1 using caspase-1-deficient BMDCs. Secretion of caspase-1 p10 and cleaved IL-1β induced by X_L_-DNA or X_S_-DNA was abolished in caspase-1-deficient dendritic cells, as shown by immunoblotting (Figure 8C). Secretion of IL-1β by X_L_-DNA or X_S_-DNA was completely abolished in caspase-1-deficient dendritic cells as determined by ELISA (Figure 8D). Poly dA:dT and ATP were positive controls to activate the inflammasome complex mediated through caspase-1 activation.

LRRFIP1 has been suggested as another cytosolic nucleic acid sensor, and so we investigated whether X-DNA could activate LRRFIP1. As a gain-of-function study, HEK293T cells were first transfected with LRRFIP1-expressing plasmid together with IFN-β-promoter reporter gene, then transfected with X_L_-DNA or poly dA:dT as a positive control. Transfection with poly dA:dT induced IFN-β expression, as IFN-β-promoter-dependent luciferase expression was increased. However, transfection with X_L_-DNA did not increase IFN-β-promoter-dependent luciferase activity (Additional file 3: Figure S3).

These results show that X_L_-DNA and X_S_-DNA induce pro-caspase-1 degradation to caspase-1 and pro-IL-1β cleavage to IL-1β when introduced into the cytosol, suggesting that both are able to activate the cytosolic inflammasome complex.

Collectively, our results demonstrate that X-shaped DNAs have immunostimulatory activity resulting in the production of immune cytokines, including IL-12, TNF-α, and IL-1β, in dendritic cells, acting as a dual activator of TLR9 and the inflammasome complex. X-shaped DNA represents a promising immune adjuvant to enhance the therapeutic efficacy of anti-cancer drugs.

## Discussion

We found that X-shaped double-stranded oligonucleotides (X-DNA) induced the activation of MAPKs and NF-κB and the expression of immune cytokines and co-stimulatory molecules in BMDCs, culminating in elevated T_H_1 cells activity. X_L_-DNA forms a complex structure through the use of a complementary sequence at each strand that results in a self-ligated X-DNA complex. Ligated X-DNA (X_L_-DNA) was more potent than single unit X-DNA (X_S_-DNA). Although the recognition of DNA by TLR9 is generally assumed, we explicitly demonstrated that the immunostimulatory activity of X-DNA was mediated through TLR9 by both gain-of-function and loss-of-function studies. We first demonstrated that X-shaped DNA directly associates with TLR9. The involvement of TLR9 in X-DNA-mediated immunostimulatory activity was further confirmed with TLR9-knockout dendritic cells. Interestingly, our results revealed that intracellular transfection of X-DNA culminated in the activation of the inflammasome complex in a caspase-1-dependent manner. These indicate a novel aspect of X-shaped DNA as a dual activator for TLR9 and inflammasomes. It has been reported that inflammasome activation in dendritic cells is critical in anti-cancer chemotherapy by linking the innate and adaptive immune responses against dying tumor cells [20]. This report showed that the lack of inflammasome components such as NLRP3 and caspase-1 results in failed activation of IFN-γ-producing CD8^+^ T cells, indicating that inflammasome activation makes anti-cancer chemotherapy against tumors more efficient. Therefore, the activation of inflammasomes by our X-DNA in dendritic cells offers an additional advantage for anti-cancer therapy compared with a single activator for TLR9.

We propose the possible therapeutic application of X_L_-DNA for anti-tumor immunotherapy, since combined therapy with X_L_-DNA enhanced the anti-tumor activity of doxorubicin in colitis-associated colon cancer progression. The anti-tumor activity of CpG-DNA has previously been shown in a mouse allograft model [18]. However, to our knowledge, this is the first study to demonstrate the anti-tumor efficacy of X_L_-DNA using a more spontaneously developed cancer model. In the future study, it would be beneficial to examine the therapeutic efficacy of X_L_-DNA in the patient-derived tumor models for preclinical and clinical applications [21].

Cancer treatment may include radiation, chemotherapy, and surgery. However, chemotherapy and radiation negatively affect normal cells. In particular, chemotherapy has dose-limiting toxic effects on cells of the immune system, which eventually leads to substantial morbidity and mortality in cancer patients [22]. The cancer immunoediting hypothesis in developing tumors, allowing evasion of host immune surveillance, has been proposed [23]. Cancer immunoediting processes consist of three distinct phases: elimination, equilibrium, and escape. Innate immune activity to block cancer progression disappears at the equilibrium phase and adaptive immune activity diminishes at the escape phase. Therefore, if immune activities can be up-regulated and maintained by immune adjuvants in cancer patients, cancer progression may be delayed. Combined therapeutic strategies designed to reactivate the patient’s own immune system to fight the tumor have been extensively pursued. As immune adjuvants for activation of innate and adaptive immune reactions, TLR agonists have come into the spotlight. A TLR9 agonist enhanced antitumor T-cell responses when used as an adjuvant for anti-cancer therapy [14]. A TLR7/8 agonist may improve cancer immunotherapy by inducing the differentiation of myeloid-derived suppressor cells, which accumulate in cancer patients and suppress the host immune system, into macrophages and dendritic cells [24]. In fact, several TLR agonists have been being developed as anti-cancer drugs in clinical trials. A TLR9 agonist of the CpG-ODN B type is being evaluated in clinical trials in patients with melanoma and lymphoma [25,26]. Our X-DNA effectively induced both innate and adaptive immune responses. X-DNA induced the activation of antigen presenting cells such as DCs to express IL-12 and co-stimulatory molecules and the differentiation of CD4^+^ T cells to T_H_1 cells to produce IFN-γ. The activation of T_H_1 cells by X-DNA was confirmed by both *in vitro* co-culture of CD4^+^ T cells and dendritic cells and *in vivo* injection into mice. Furthermore, inflammasomes were also activated by X-DNA to secrete IL-1β. These results suggest that X-DNA is an excellent immune adjuvant that can be used in combination therapy with anti-cancer drugs, reducing the required dosage of doxorubicin and thereby possibly alleviating side effects of anti-cancer drugs. Our results indicate a promising new candidate of an immune adjuvant for anti-cancer immunotherapy, especially for colon cancer.

## Conclusions

X_L_-DNA and X_S_-DNA had immunostimulatory activity via the dual activation of TLR9 and inflammasomes in dendritic cells, leading to T cell activation. X_L_-DNA was effective as an immune adjuvant, enhancing the therapeutic efficacy of an anti-cancer drug in a colitis-associated colon cancer animal model.

## Methods

### Animals and cell culture

Animal care and the study protocols were carried out in accordance with the guidelines of the Institutional Animal Care and Use Committee (IACUC) of the Catholic University of Korea (permission # 2012-5-001). C57BL/6 and Balb/C mice were purchased from Orient Bio (Seoul, Korea) and were acclimated under specific pathogen-free conditions in an animal facility for at least a week before experiments. The mice were housed in a temperature (23 ± 3°C) and relative humidity (40-60%)-controlled room. Balb/C TLR9 knockout (KO) mice were obtained from Hyung-Joo Kwon (Hallym University, Gangwon-do, Korea). C57BL/6 caspase-1KO mice were purchased from the Jackson Laboratory (Bar Harbor, ME, USA).

To prepare conventional dendritic cells (cDCs), bone marrow cells were isolated from mice and cultured in RPMI1640 medium containing 10% (v/v) heat-inactivated fetal bovine serum (Life Technologies; Grand Island, NY, USA), 50 μM of 2-mercaptoethanol, 100 units/ml of penicillin, 100 μg/ml of streptomycin, 2 mM of glutamine, and 3% J558L hybridoma cell culture supernatant containing granulocyte-macrophage colony-stimulating factor (GM-CSF) for 6 days. Non-adherent cells were used as dendritic cells (DCs) [27].

HEK293T cells (human embryonic kidney cells) were cultured in Dulbecco’s modified eagle medium supplemented with 10% fetal bovine serum, 100 units/ml of penicillin, and 100 μg/ml of streptomycin. Cells were maintained at 37°C in a 5% CO_2_/air environment [28].

### Reagents

X-shape double-stranded oligodeoxynucleotides (X-DNA) were prepared as described previously [11]. X_L_-DNA refers to X-DNA forming a ligated complex through self-ligation due to ACGT sequences at the end of the strands, while X_S_-DNA refers to X-DNA existing as a single module due to the lack of sticky ACGT sequences. Sequences of X-DNAs are shown in Additional file 1: Figure S1. ODN1668 was purchased from TIB MOLBIOL (Berlin, Germany). R848 was purchased from Enzo Life Sciences (Farmingdale, NY, USA). Poly dA:dT and dynasore were obtained from Sigma Aldrich (St. Louis, MO, USA). Bafilomycin A1 was purchased from EMD Millipore (Billerica, MA, USA). ATP was purchased from InvivoGen (San Diego, CA, USA). Ovalbumin (323-339) peptide was purchased from AnaSpec (San Jose, CA, USA).

### Immunoblotting

Immunoblotting was performed using SDS-PAGE as previously described [29]. Antibodies against phospho-ERK, phospho-JNK, phospho-p38, phospho-IκBα, ERK, JNK, p38, and actin were purchased from Cell Signaling Technology (Boston, MA, USA). Antibody against capase-1 was obtained from Santa Cruz Biotechnology (Dallas, TX, USA). Antibody against IL-1β was purchased from R&D Systems (Minneapolis, MN, USA)

### Reverse transcription (RT) and quantitative PCR analysis

PCR was performed as previously described [30]. Total RNAs were isolated with Welprep™ reagent (Jeil Biotechservices Inc., Daegu, Korea). RNAs were reverse-transcribed with ImProm-II™ Reverse Transcriptase (Promega, Madison, WI, USA) and amplified with IQ™ SYBR® Green Supermix (Bio-Rad, Hercules, CA, USA) using an IQ™5 (Bio-Rad) for quantitative real-time PCR. The primers were: *Il-12*, 5′-GAAGTTCAACATCAAGAGCAGTAG-3′ and 5′-AGGGAGAAGTAGGAATGGGG-3′; *Tnf-α*, 5′-AAAATTCGAGTGACAAGCCTGTAG-3′ and 5′-CCCTTGAAGAGAACCTGGGAGTAG-3′; *Ifn-β*, 5′-TCCAAGAAAGGACGAACATTCG-3′ and 5′-TGAGGACATCTCCCACGTCAA-3′; *β-actin*, 5′-TCATGAAGTGTGACGTTGACATCCGT-3′ and 5′-TTGCGGTGCACGATGGAGGGGCCGGA-3′. The specificity of the amplified PCR products was assessed by a melting curve analysis. Fold-induction of gene expression was calculated after mRNA levels of each target gene were normalized to β-actin levels in corresponding samples.

### Enzyme-linked immunosorbent assay (ELISA)

ELISA was performed as previously described [31]. Concentrations of IL-12 p40 (eBioscience Inc., San Diego, CA, USA), TNF-α (R&D Systems), IFN-γ (eBioscience Inc.), and IL-1β (R&D Systems) in the culture supernatants were determined by ELISA according to the manufacturer’s instructions. Plates were read at 450 nm wavelength with a microplate reader (Molecular Devices, San Francisco, CA, USA). The concentration ranges of the standard curves were 19.5 to 20,000, 9.7 to 10,000, 31.25 to 4,000, and 31.25 to 2,000 pg/ml for IL-12 p40, TNF-α, IFN-γ, and IL-1β, respectively. Samples were properly diluted to be measured within the standard curve ranges.

### Flow cytometric analysis

After BMDCs were treated with X-DNAs or CpG 1668 for 24 h, CD80 and CD86 surface molecules were stained with FITC-conjugated anti-mouse antibodies against CD80 and CD86 (BD Pharmingen, San Jose, CA, USA) for 3 h at 4°C. After washing with staining buffer (BD Pharmingen), cells were analyzed by FACSCanto flow cytometer (BD Biosciences, San Jose, CA, USA).

### Assay for T cell activation by DCs

T_H_ cells were isolated from lymph nodes of C57BL/6 mice using anti-mouse CD4 microbeads and MACS LS columns according to the manufacturer’s protocol (Miltenyi Biotec, Auburn, WA, USA). After BMDCs were stimulated with ovalbumin (323-339) (5 μg/ml) in the presence or absence of X_L_-DNA for 24 h, cells were co-cultured with T_H_ cells for 5 days. The concentrations of cytokines in the culture supernatants were determined by ELISA.

### In vivo immunostimulatory activity of X_L_-DNA

X_L_-DNA (20 nmol) or ODN1668 (20 nmol) was intraperitoneally injected into Balb/C mice. After 3 h, blood samples were obtained by eye bleeding and the concentrations of cytokines were determined by ELISA.

### Animal model of colitis-associated colon cancer

A colitis-associated cancer model was induced as previously described with slight modifications [32]. Colitis-associated cancer was induced by a single intraperitoneal injection of a mutagenic agent, azoxymethane (AOM, 10 mg/kg; Sigma-Aldrich) into Balb/C mice on day 1 followed by 2 cycles of 2% dextran sulfate sodium (DSS) in drinking water for 1 week and normal drinking water for 1 week. The mice were divided into six groups (n = 10-12/group) and intravenously injected with doxorubicin (1 or 2 mg/kg) with or without X_L_-DNA twice per week for 3 weeks. The mice were sacrificed and their colons were removed. The number and the size of polyps that were 3 mm or larger were determined from each mouse. For histological examination, colon tissues were infused with 4% paraformaldehyde and embedded in paraffin. Sections from these samples were stained with hematoxylin and eosin.

### Transfection and luciferase assay

A NF-κB (2x)-luciferase reporter plasmid and pMSCV puro-mTLR9-Myc-expressing plasmid for luciferase assay were provided by Youme Kim (POSTECH, Pohang, Korea). LRRFIP1-expressing plasmid was provided by Xuetao Cao (Second Military Medical University, Shanghai, China). IFN-β-luciferase plasmid was provided by Shizuo Akira (Osaka University, Osaka, Japan). Transfection of plasmids and measurement of luciferase activity were as described previously [31].

### Immunoprecipitation study for X_L_-DNA binding to TLR9

Immunoprecipitation was performed as previously described [33]. HEK293T cells were transfected with the expression plasmid of pcDNA3.1-mTLR9-Myc/His. Cell lysates were incubated with biotinylated X_L_-DNA for 2 h and immunoprecipitated with NeutrAvidin(NA)-beads (Thermo Scientific, Rockford, IL, USA) for 4 h at 4°C on a rocker. Immune complexes were solubilized with Laemmli sample buffer after washing three times. The solubilized proteins were resolved on SDS-PAGE and immunoblotting was performed. Anti-Myc antibody was purchased from Cell Signaling Technology.

### Statistical analysis

Data are expressed as mean ± SEM. Comparisons of data between groups were examined by one-way ANOVA followed by Tukey’s multiple range test (significant when *p* < 0.05).

## Additional files

Additional file 1: Figure S1. The structure and sequences of X-shaped double-stranded DNA (X-DNA). X_L_-DNA forms a ligated complex due to an ACGT sequence (bolded) at the end of the strand. X_S_-DNA does not have the ACGT sequence and exists as a single module. Additional file 2: Figure S2. X_S_-DNA-induced activation of dendritic cells is TLR9-dependent. BMDCs from wild-type (WT) or TLR9-knockout (KO) mice were treated with X_S_-DNA (1 μM) for (A) 4 h and (B) 18 h. For A, mRNA levels were determined by quantitative real time-PCR. For B, concentrations of cytokines were measured by ELISA. Values are mean ± SEM (n = 3). *, *p* < 0.05. Veh, vehicle. Representative data from three independent experiments are presented. Additional file 3: Figure S3. X_L_-DNA is unable to activate LRRFIP1. HEK293T cells were transfected with pCMV-LRRFIP1-expressing plasmid, a luciferase reporter plasmid containing IFN-β-promotor, and β-galactosidase expression vector. Cells were transfected with X_L_-DNA or poly dA:dT for 8 h. Luciferase activities were normalized by β-galactosidase activities and are presented as fold inductions. Values are mean ± SEM (n = 3). *, *p* < 0.05. Representative data from three independent experiments are presented.
