# Supplementary figures and images for: X-shaped DNA potentiates therapeutic efficacy in colitis-associated colon cancer through dual activation of TLR9 and inflammasomes
Source: Mol Cancer. 2015 May 15;14:104. doi: 10.1186/s12943-015-0369-2 (PMC4431032; doi:10.1186/s12943-015-0369-2)

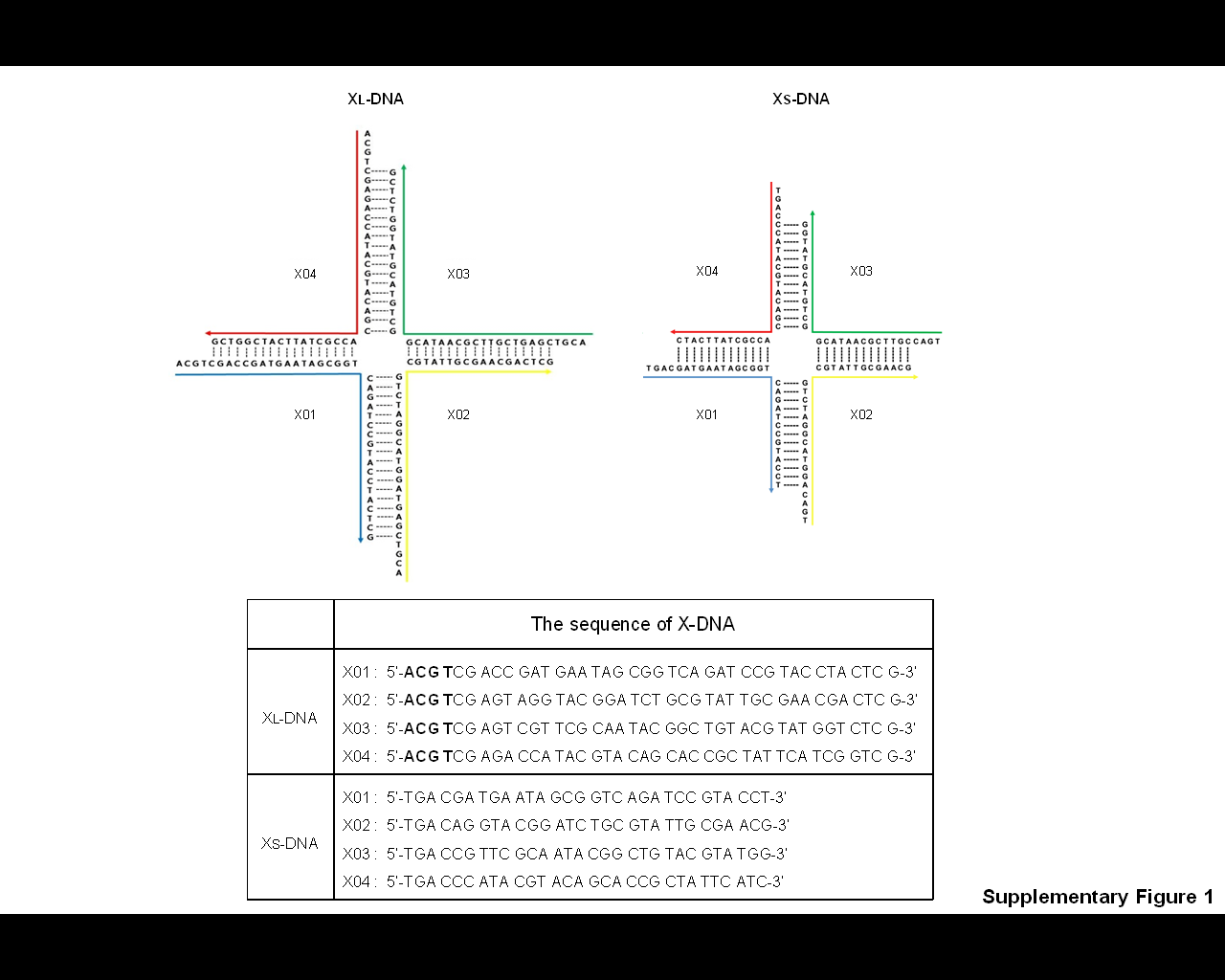

Supplement: Additional file 1: Figure S1. — The structure and sequences of X-shaped double-stranded DNA (X-DNA). XL-DNA forms a ligated complex due to an ACGT sequence (bolded) at the end of the strand. XS-DNA does not have the ACGT sequence and exists as a single module. [file 12943_2015_369_MOESM1_ESM.doc]

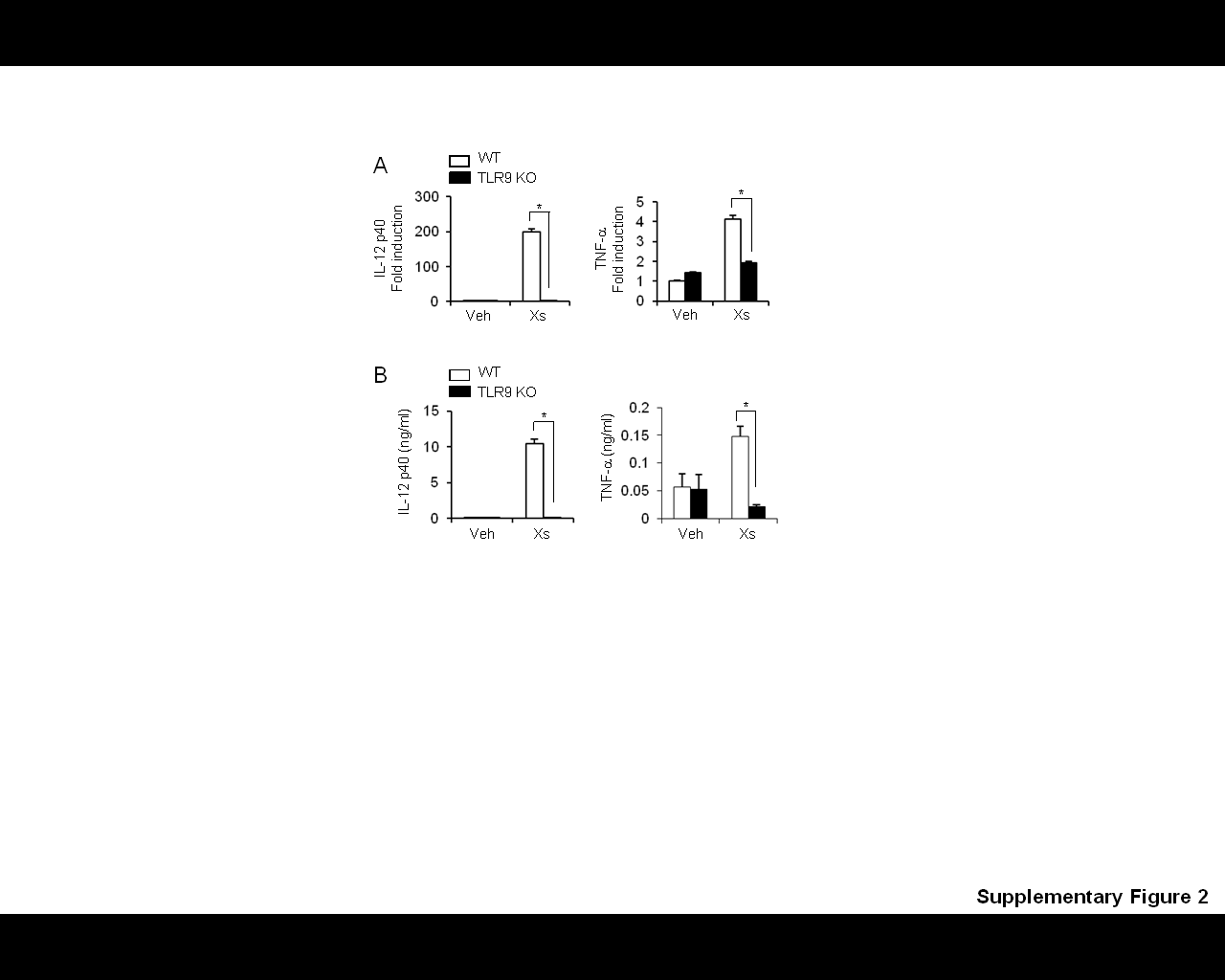

Supplement: Additional file 2: Figure S2. — XS-DNA-induced activation of dendritic cells is TLR9-dependent. BMDCs from wild-type (WT) or TLR9-knockout (KO) mice were treated with XS-DNA (1 μM) for (A) 4 h and (B) 18 h. For A, mRNA levels were determined by quantitative real time-PCR. For B, concentrations of cytokines were measured by ELISA. Values are mean ± SEM (n = 3). *, p < 0.05. Veh, vehicle. Representative data from three independent experiments are presented. [file 12943_2015_369_MOESM2_ESM.doc]

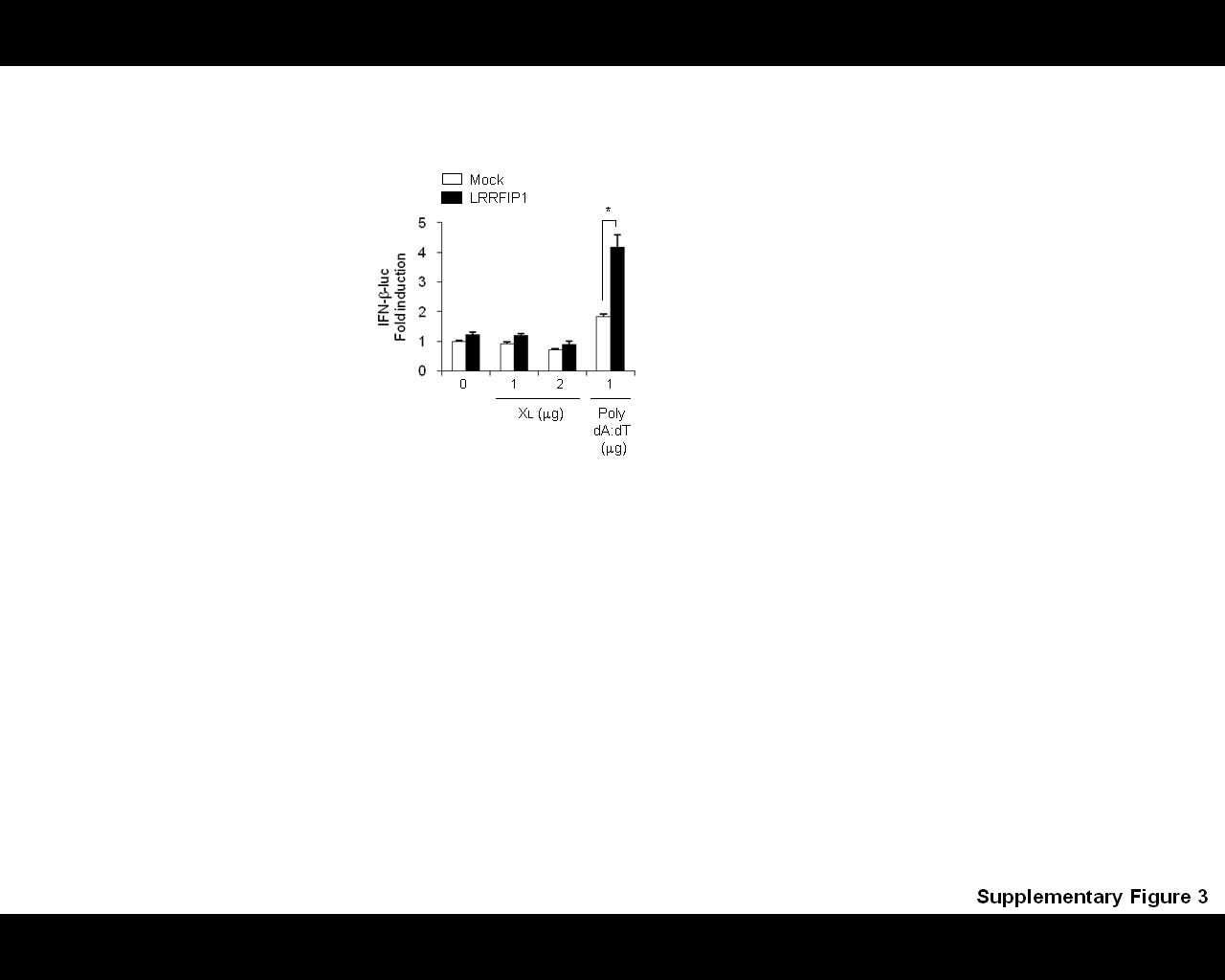

Supplement: Additional file 3: Figure S3. — XL-DNA is unable to activate LRRFIP1. HEK293T cells were transfected with pCMV-LRRFIP1-expressing plasmid, a luciferase reporter plasmid containing IFN-β-promotor, and β-galactosidase expression vector. Cells were transfected with XL-DNA or poly dA:dT for 8 h. Luciferase activities were normalized by β-galactosidase activities and are presented as fold inductions. Values are mean ± SEM (n = 3). *, p < 0.05. Representative data from three independent experiments are presented. [file 12943_2015_369_MOESM3_ESM.doc]
